# The University of Michigan

**Published:** 1856-05

**Authors:** 


*The University of Michigan.*—The alternative mandamus granted by the Supreme Court, requiring the Regents of the University to show cause why a Professor of Homœopathy has not been appointed, in compliance with the Act of the Legislature requiring such appointment, has been overruled in the same court, on the answer of the Regents.

The "Peninsular Journal," for April, has the argument of the counsel of the Regents, and a brief editorial commending it especially to our attention. We have accordingly given it a perusal, and find only one thing to regret, viz., that it does not give, except in general terms, the decision of the court.

Will the "Peninsular" pardon us if we importune it with questions to which we have no right to claim an answer, though we might be much enlightened thereby.

Did the court decide that the Legislature had no power to create such a chair?

Did it decide that the public good does not require such a chair, and that the homœopaths have no right to representation in the schools?

Or did it simply decide that Mr. Elijah H. Drake had mistaken his mission, and was unnecessarily patriotic in relieving the Attorney General of his official duties?

And, finally, will the "Peninsular" believe us, when we express the cordial wish that no Professorship of Homœopathy may ever be established in the University of Michigan?

The "Peninsular," as well as every friend of legitimate medicine, has a right to rejoice in the decision on any one of these three grounds; but it would be much more satisfactory to know that the *whole* question had been decided, and that the foolish and insane project of homœopathic quackery was effectually killed. We have, however, much confidence in the courts—

there is a natural tendency on the part of the legal profession to recognize the claims of "regular" medicine as the true and only representative of medical science. We trust that the Bar of Michigan may stand between its University and the invasion of quackery. If it does not, let our friends then make an earnest move to have a Professor of Fourierite or Communistic Law established, and bring them to their senses.

But we beg not to be understood as in any way approving of the system of government medical education. We deny the right of any government to tax its people, directly or indirectly, for the purposes of education, farther than the safety of the commonwealth may require; we deny the policy of a gratuitous education to a profession of gentlemen; we deny the safety of entrusting the cause of medical education to any authority outside of the profession; and we deprecate the importation of European systems (founded on the admission of the relation of serf and master) to the free soil of our country.

This is our creed. We have yet to see the first solid argument advanced against it.

